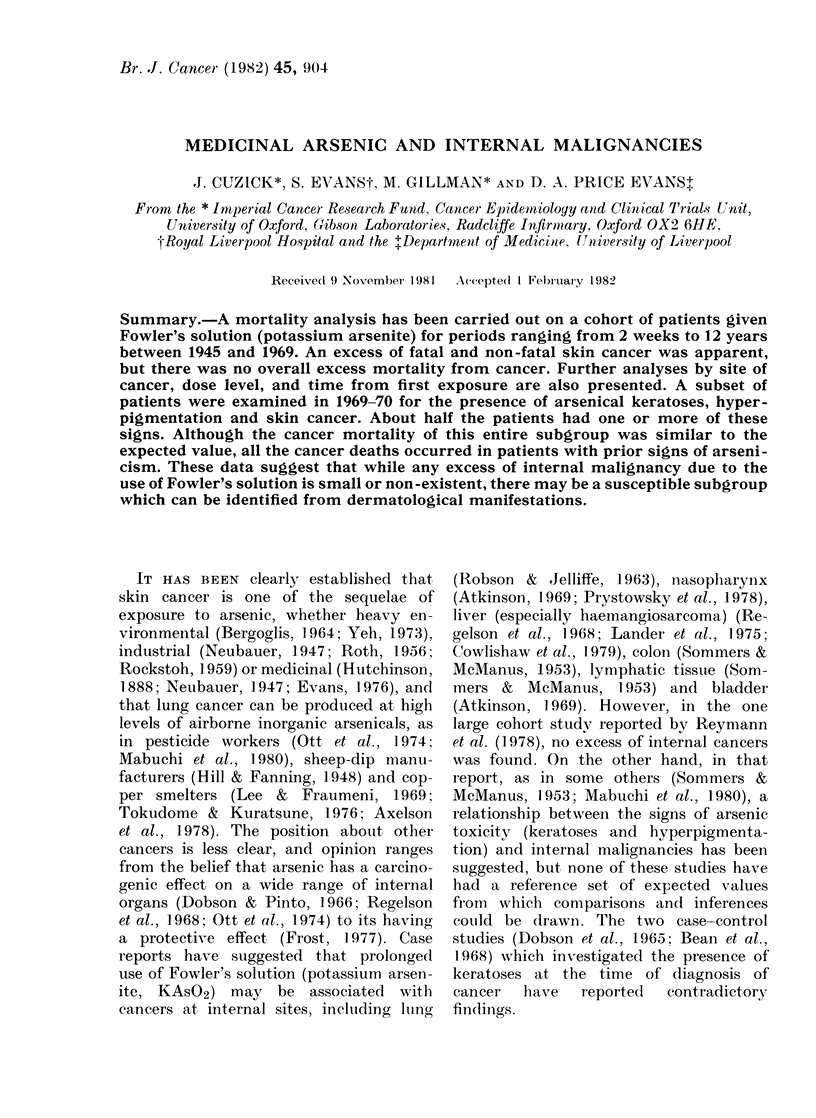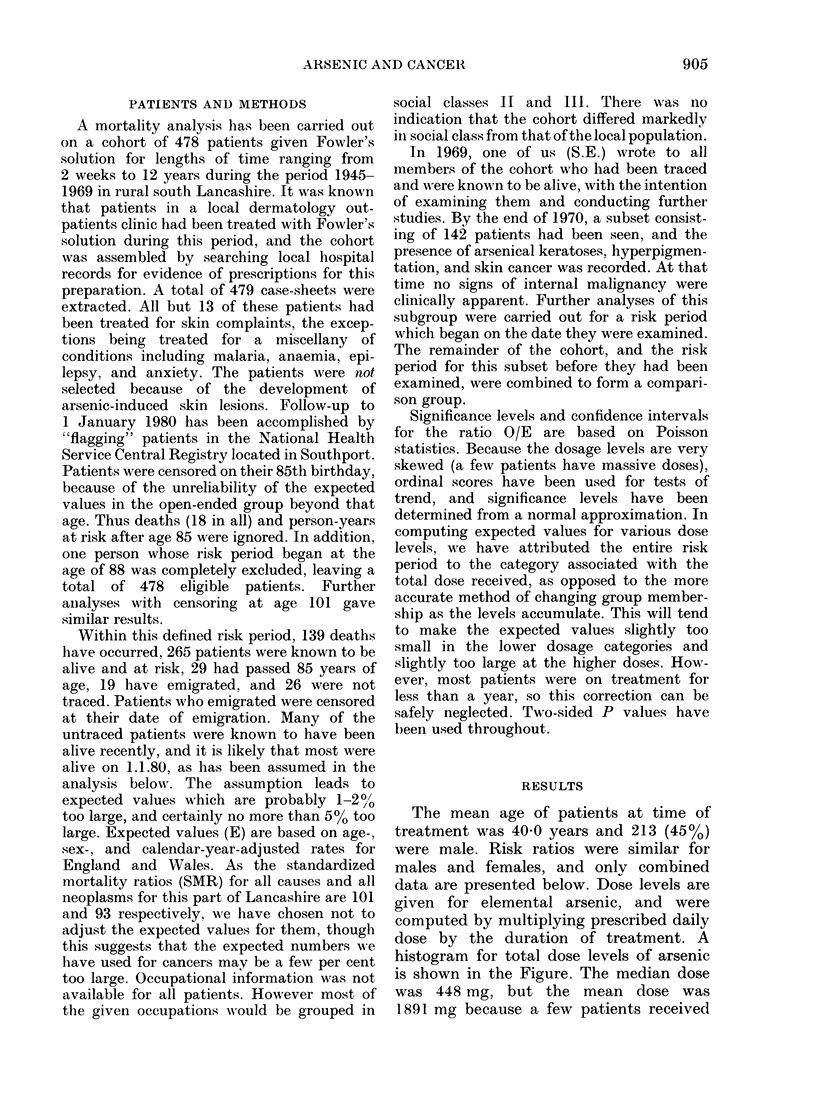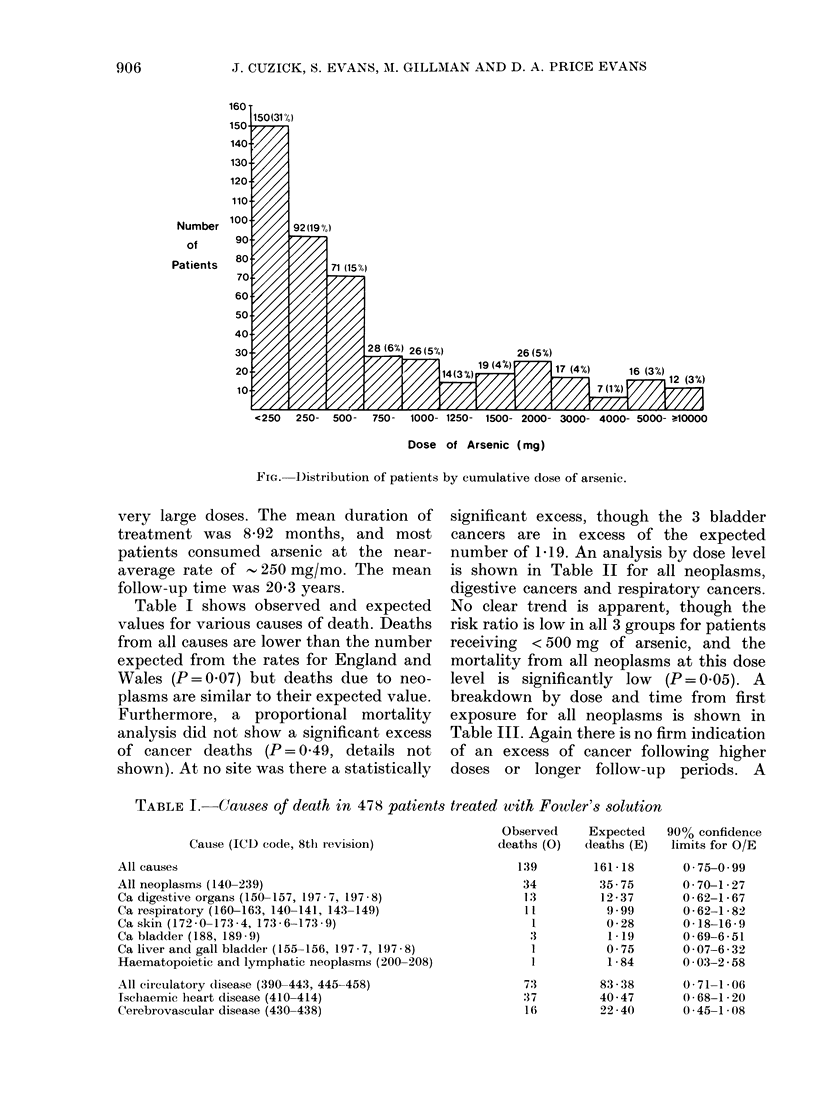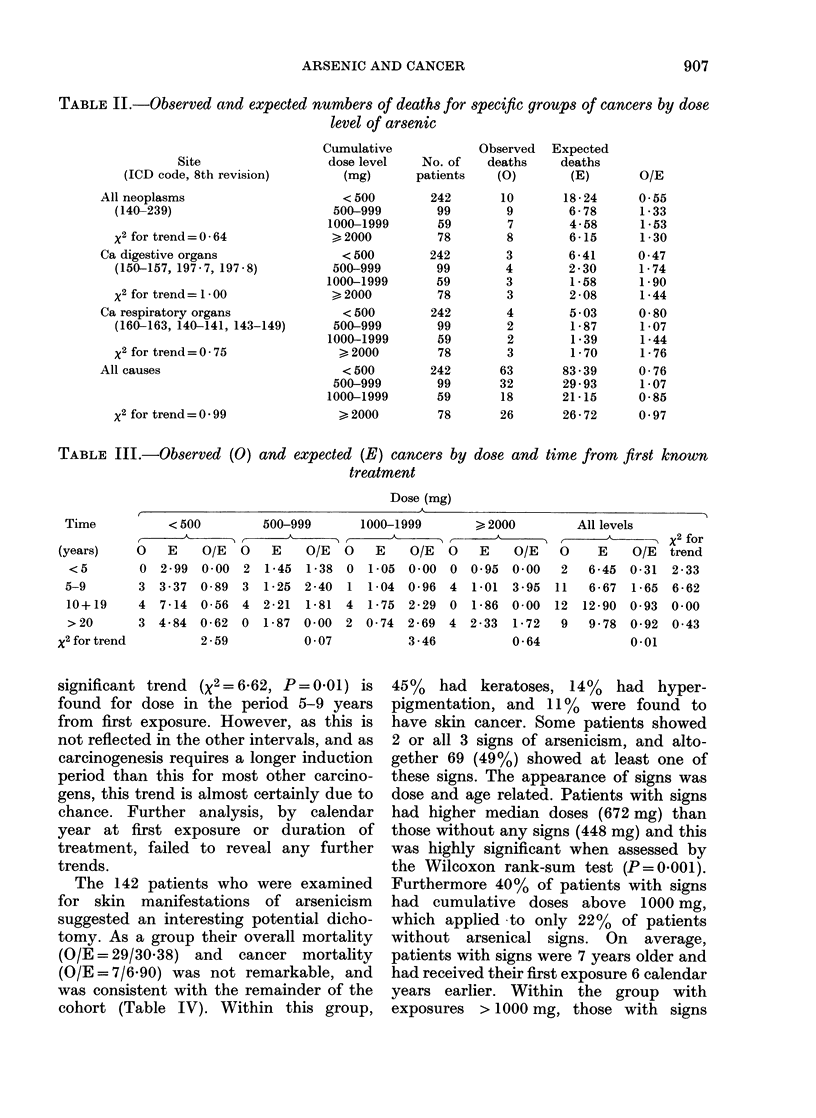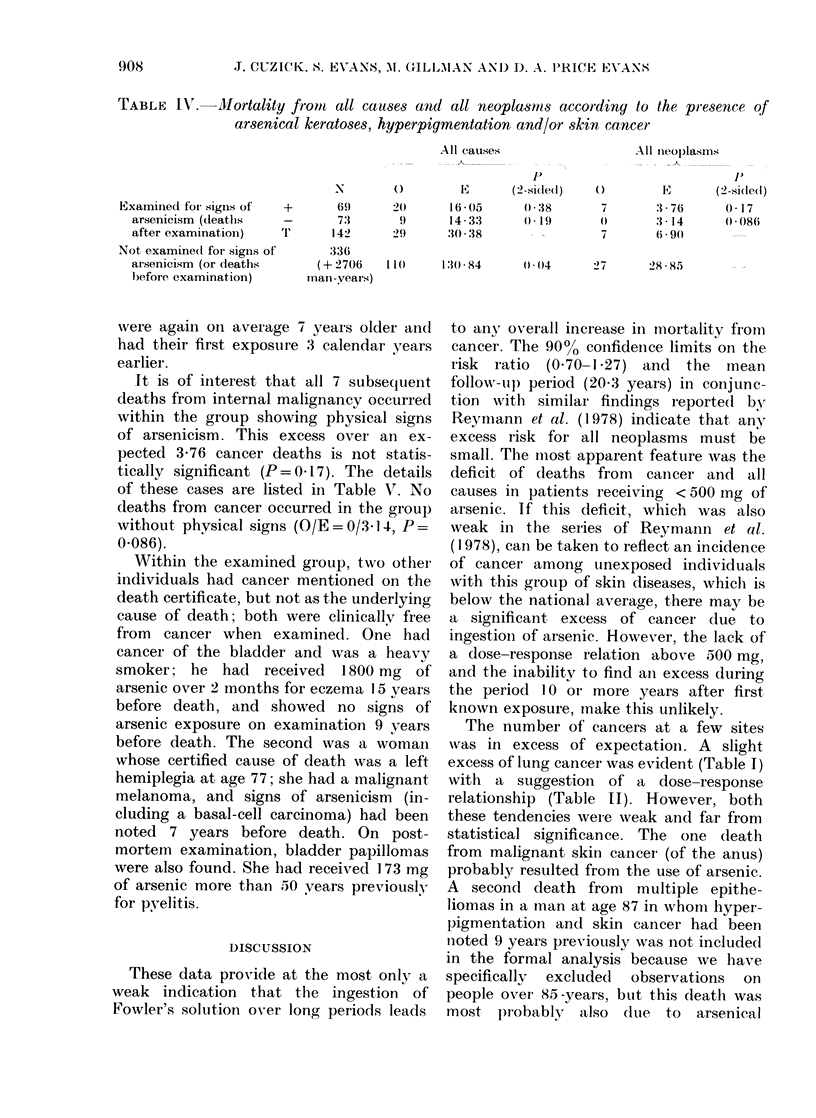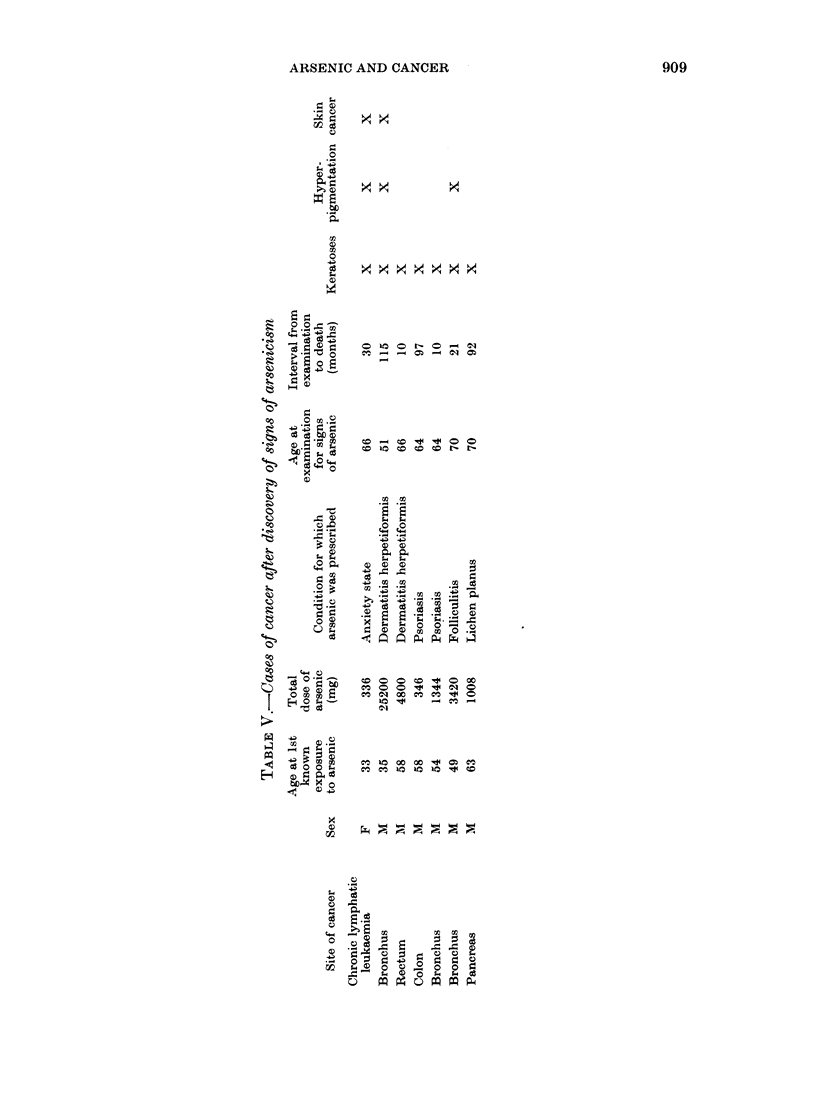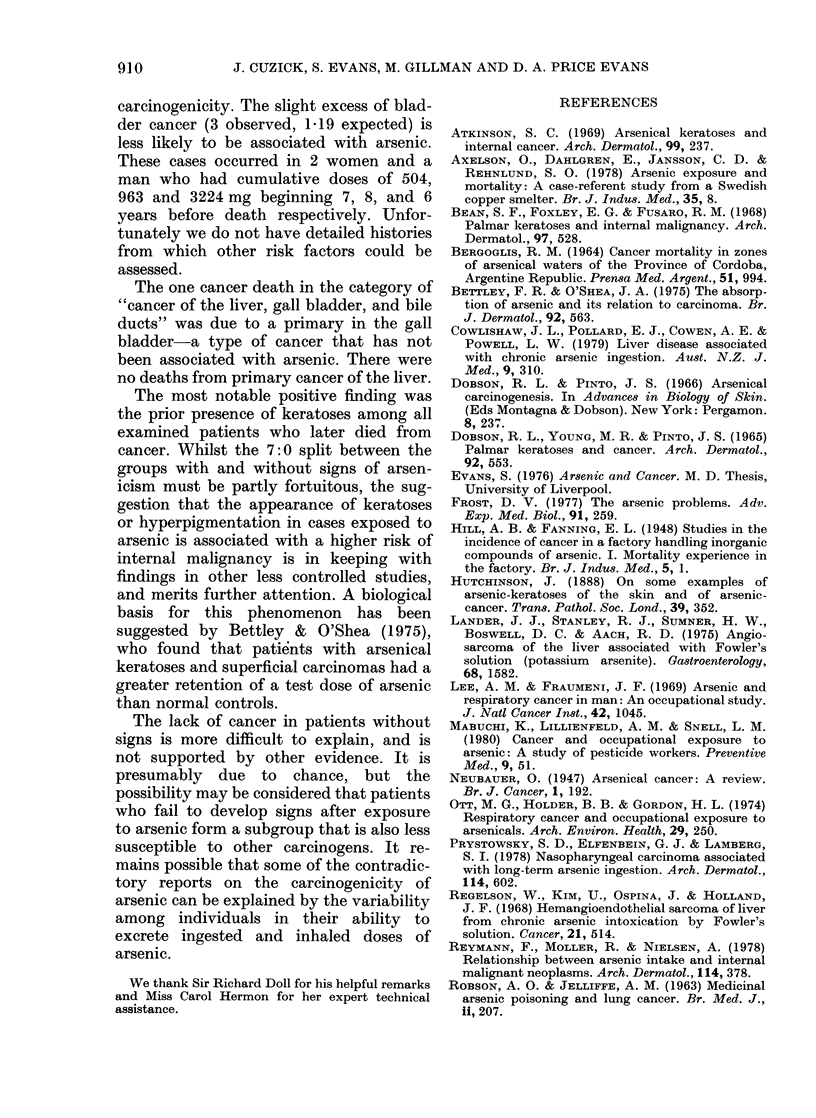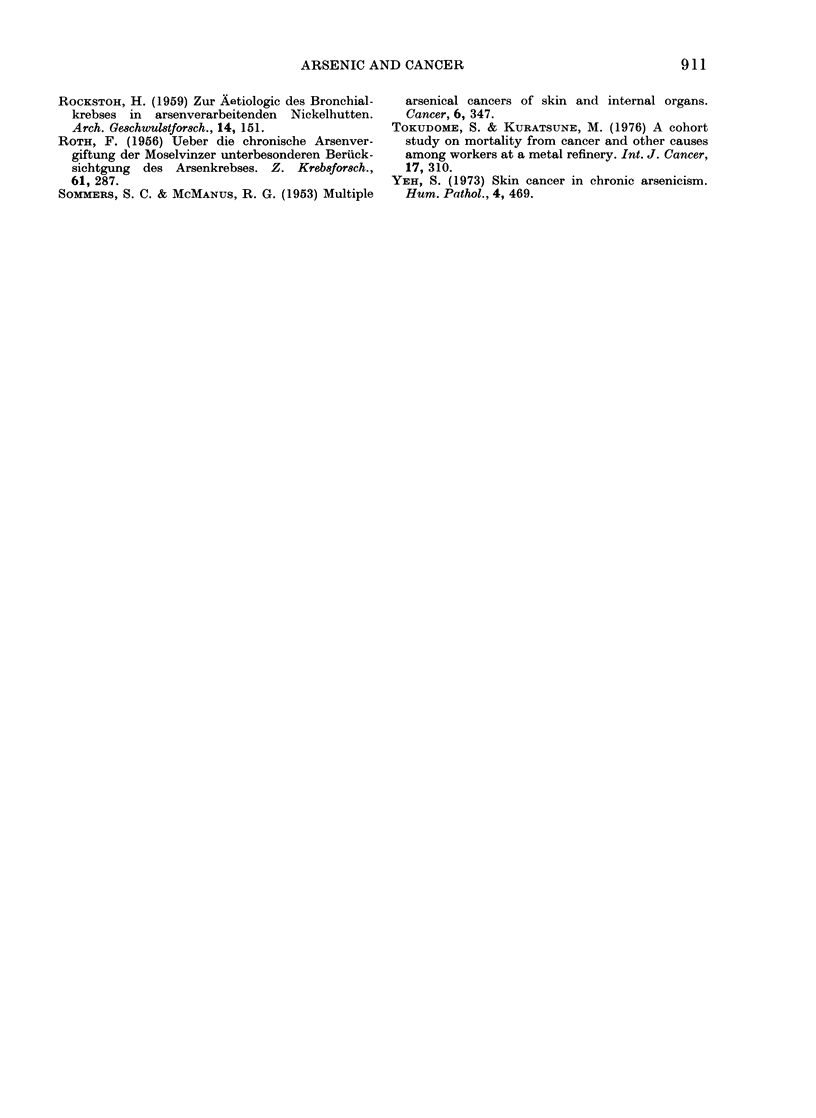# Medicinal arsenic and internal malignancies.

**DOI:** 10.1038/bjc.1982.143

**Published:** 1982-06

**Authors:** J. Cuzick, S. Evans, M. Gillman, D. A. Price Evans

## Abstract

A mortality analysis has been carried out on a cohort of patients given Fowler's solution (potassium arsenite) for periods ranging from 2 weeks to 12 years between 1945 and 1969. An excess of fatal and non-fatal skin cancer was apparent, but there was no overall excess mortality from cancer. Further analyses by site of cancer, dose level, and time from first exposure are also presented. A subset of patients were examined in 1969-70 for the presence of arsenical keratoses, hyperpigmentation and skin cancer. About half the patients had one or more of these signs. Although the cancer mortality of this entire subgroup was similar to the expected value, all the cancer deaths occurred in patients with prior signs of arsenicism. These data suggest that while any excess of internal malignancy due to the use of Fowler's solution is small or non-existent, there may be a susceptible subgroup which can be identified from dermatological manifestations.


*Br. J. Cancer* (1982) **45**, 904

### MEDICINAL ARSENIC AND INTERNAL MALIGNANCIES

J. CUZICK*, S. EVANS†, M. GILLMAN* AND D. A. PRICE EVANS‡

*From the * Imperial Cancer Research Fund, Cancer Epidemiology and Clinical Trials Unit, University of Oxford, Gibson Laboratories, Radcliffe Infirmary, Oxford OX2 6HE, †Royal Liverpool Hospital and the ‡Department of Medicine, University of Liverpool*

Received 9 November 1981 Accepted 1 February 1982

**Summary.—A mortality analysis has been carried out on a cohort of patients given Fowler's solution (potassium arsenite) for periods ranging from 2 weeks to 12 years between 1945 and 1969. An excess of fatal and non-fatal skin cancer was apparent, but there was no overall excess mortality from cancer. Further analyses by site of cancer, dose level, and time from first exposure are also presented. A subset of patients were examined in 1969–70 for the presence of arsenical keratoses, hyperpigmentation and skin cancer. About half the patients had one or more of these signs. Although the cancer mortality of this entire subgroup was similar to the expected value, all the cancer deaths occurred in patients with prior signs of arsenicism. These data suggest that while any excess of internal malignancy due to the use of Fowler's solution is small or non-existent, there may be a susceptible subgroup which can be identified from dermatological manifestations.**

IT HAS BEEN clearly established that skin cancer is one of the sequelae of exposure to arsenic, whether heavy environmental (Bergoglis, 1964; Yeh, 1973), industrial (Neubauer, 1947; Roth, 1956; Rockstoh, 1959) or medicinal (Hutchinson, 1888; Neubauer, 1947; Evans, 1976), and that lung cancer can be produced at high levels of airborne inorganic arsenicals, as in pesticide workers (Ott *et al.*, 1974; Mabuchi *et al.*, 1980), sheep-dip manufacturers (Hill & Fanning, 1948) and copper smelters (Lee & Fraumeni, 1969; Tokudome & Kuratsune, 1976; Axelson *et al.*, 1978). The position about other cancers is less clear, and opinion ranges from the belief that arsenic has a carcinogenic effect on a wide range of internal organs (Dobson & Pinto, 1966; Regelson *et al.*, 1968; Ott *et al.*, 1974) to its having a protective effect (Frost, 1977). Case reports have suggested that prolonged use of Fowler's solution (potassium arsenite, $KAsO_2$) may be associated with cancers at internal sites, including lung (Robson & Jelliffe, 1963), nasopharynx (Atkinson, 1969; Prystowsky *et al.*, 1978), liver (especially haemangiosarcoma) (Regelson *et al.*, 1968; Lander *et al.*, 1975; Cowlishaw *et al.*, 1979), colon (Sommers & McManus, 1953), lymphatic tissue (Sommers & McManus, 1953) and bladder (Atkinson, 1969). However, in the one large cohort study reported by Reymann *et al.* (1978), no excess of internal cancers was found. On the other hand, in that report, as in some others (Sommers & McManus, 1953; Mabuchi *et al.*, 1980), a relationship between the signs of arsenic toxicity (keratoses and hyperpigmentation) and internal malignancies has been suggested, but none of these studies have had a reference set of expected values from which comparisons and inferences could be drawn. The two case–control studies (Dobson *et al.*, 1965; Bean *et al.*, 1968) which investigated the presence of keratoses at the time of diagnosis of cancer have reported contradictory findings.

### PATIENTS AND METHODS

A mortality analysis has been carried out on a cohort of 478 patients given Fowler's solution for lengths of time ranging from 2 weeks to 12 years during the period 1945–1969 in rural south Lancashire. It was known that patients in a local dermatology out-patients clinic had been treated with Fowler's solution during this period, and the cohort was assembled by searching local hospital records for evidence of prescriptions for this preparation. A total of 479 case-sheets were extracted. All but 13 of these patients had been treated for skin complaints, the exceptions being treated for a miscellany of conditions including malaria, anaemia, epilepsy, and anxiety. The patients were *not* selected because of the development of arsenic-induced skin lesions. Follow-up to 1 January 1980 has been accomplished by "flagging" patients in the National Health Service Central Registry located in Southport. Patients were censored on their 85th birthday, because of the unreliability of the expected values in the open-ended group beyond that age. Thus deaths (18 in all) and person-years at risk after age 85 were ignored. In addition, one person whose risk period began at the age of 88 was completely excluded, leaving a total of 478 eligible patients. Further analyses with censoring at age 101 gave similar results.

Within this defined risk period, 139 deaths have occurred, 265 patients were known to be alive and at risk, 29 had passed 85 years of age, 19 have emigrated, and 26 were not traced. Patients who emigrated were censored at their date of emigration. Many of the untraced patients were known to have been alive recently, and it is likely that most were alive on 1.1.80, as has been assumed in the analysis below. The assumption leads to expected values which are probably 1–2% too large, and certainly no more than 5% too large. Expected values (E) are based on age-, sex-, and calendar-year-adjusted rates for England and Wales. As the standardized mortality ratios (SMR) for all causes and all neoplasms for this part of Lancashire are 101 and 93 respectively, we have chosen not to adjust the expected values for them, though this suggests that the expected numbers we have used for cancers may be a few per cent too large. Occupational information was not available for all patients. However most of the given occupations would be grouped in social classes II and III. There was no indication that the cohort differed markedly in social class from that of the local population.

In 1969, one of us (S.E.) wrote to all members of the cohort who had been traced and were known to be alive, with the intention of examining them and conducting further studies. By the end of 1970, a subset consisting of 142 patients had been seen, and the presence of arsenical keratoses, hyperpigmentation, and skin cancer was recorded. At that time no signs of internal malignancy were clinically apparent. Further analyses of this subgroup were carried out for a risk period which began on the date they were examined. The remainder of the cohort, and the risk period for this subset before they had been examined, were combined to form a comparison group.

Significance levels and confidence intervals for the ratio O/E are based on Poisson statistics. Because the dosage levels are very skewed (a few patients have massive doses), ordinal scores have been used for tests of trend, and significance levels have been determined from a normal approximation. In computing expected values for various dose levels, we have attributed the entire risk period to the category associated with the total dose received, as opposed to the more accurate method of changing group membership as the levels accumulate. This will tend to make the expected values slightly too small in the lower dosage categories and slightly too large at the higher doses. However, most patients were on treatment for less than a year, so this correction can be safely neglected. Two-sided $P$ values have been used throughout.

### RESULTS

The mean age of patients at time of treatment was 40·0 years and 213 (45%) were male. Risk ratios were similar for males and females, and only combined data are presented below. Dose levels are given for elemental arsenic, and were computed by multiplying prescribed daily dose by the duration of treatment. A histogram for total dose levels of arsenic is shown in the Figure. The median dose was 448 mg, but the mean dose was 1891 mg because a few patients received

FIG.—Distribution of patients by cumulative dose of arsenic.

very large doses. The mean duration of treatment was 8·92 months, and most patients consumed arsenic at the near-average rate of ~250 mg/mo. The mean follow-up time was 20·3 years.

Table I shows observed and expected values for various causes of death. Deaths from all causes are lower than the number expected from the rates for England and Wales ($P = 0{\cdot}07$) but deaths due to neoplasms are similar to their expected value. Furthermore, a proportional mortality analysis did not show a significant excess of cancer deaths ($P = 0{\cdot}49$, details not shown). At no site was there a statistically significant excess, though the 3 bladder cancers are in excess of the expected number of 1·19. An analysis by dose level is shown in Table II for all neoplasms, digestive cancers and respiratory cancers. No clear trend is apparent, though the risk ratio is low in all 3 groups for patients receiving < 500 mg of arsenic, and the mortality from all neoplasms at this dose level is significantly low ($P = 0{\cdot}05$). A breakdown by dose and time from first exposure for all neoplasms is shown in Table III. Again there is no firm indication of an excess of cancer following higher doses or longer follow-up periods. A

TABLE I.—*Causes of death in 478 patients treated with Fowler's solution*

| Cause (ICD code, 8th revision) | Observed deaths (O) | Expected deaths (E) | 90% confidence limits for O/E |
|---|---|---|---|
| All causes | 139 | 161·18 | 0·75–0·99 |
| All neoplasms (140–239) | 34 | 35·75 | 0·70–1·27 |
| Ca digestive organs (150–157, 197·7, 197·8) | 13 | 12·37 | 0·62–1·67 |
| Ca respiratory (160–163, 140–141, 143–149) | 11 | 9·99 | 0·62–1·82 |
| Ca skin (172·0–173·4, 173·6–173·9) | 1 | 0·28 | 0·18–16·9 |
| Ca bladder (188, 189·9) | 3 | 1·19 | 0·69–6·51 |
| Ca liver and gall bladder (155–156, 197·7, 197·8) | 1 | 0·75 | 0·07–6·32 |
| Haematopoietic and lymphatic neoplasms (200–208) | 1 | 1·84 | 0·03–2·58 |
| All circulatory disease (390–443, 445–458) | 73 | 83·38 | 0·71–1·06 |
| Ischaemic heart disease (410–414) | 37 | 40·47 | 0·68–1·20 |
| Cerebrovascular disease (430–438) | 16 | 22·40 | 0·45–1·08 |

TABLE II.—*Observed and expected numbers of deaths for specific groups of cancers by dose level of arsenic*

| Site (ICD code, 8th revision) | Cumulative dose level (mg) | No. of patients | Observed deaths (O) | Expected deaths (E) | O/E |
|---|---|---|---|---|---|
| All neoplasms | < 500 | 242 | 10 | 18·24 | 0·55 |
| (140–239) | 500–999 | 99 | 9 | 6·78 | 1·33 |
| | 1000–1999 | 59 | 7 | 4·58 | 1·53 |
| $\chi^2$ for trend = 0·64 | ⩾ 2000 | 78 | 8 | 6·15 | 1·30 |
| Ca digestive organs | < 500 | 242 | 3 | 6·41 | 0·47 |
| (150–157, 197·7, 197·8) | 500–999 | 99 | 4 | 2·30 | 1·74 |
| | 1000–1999 | 59 | 3 | 1·58 | 1·90 |
| $\chi^2$ for trend = 1·00 | ⩾ 2000 | 78 | 3 | 2·08 | 1·44 |
| Ca respiratory organs | < 500 | 242 | 4 | 5·03 | 0·80 |
| (160–163, 140–141, 143–149) | 500–999 | 99 | 2 | 1·87 | 1·07 |
| | 1000–1999 | 59 | 2 | 1·39 | 1·44 |
| $\chi^2$ for trend = 0·75 | ⩾ 2000 | 78 | 3 | 1·70 | 1·76 |
| All causes | < 500 | 242 | 63 | 83·39 | 0·76 |
| | 500–999 | 99 | 32 | 29·93 | 1·07 |
| | 1000–1999 | 59 | 18 | 21·15 | 0·85 |
| $\chi^2$ for trend = 0·99 | ⩾ 2000 | 78 | 26 | 26·72 | 0·97 |

TABLE III.—*Observed (O) and expected (E) cancers by dose and time from first known treatment*

| Time | Dose (mg) < 500 | | | 500–999 | | | 1000–1999 | | | ⩾ 2000 | | | All levels | | | $\chi^2$ for trend |
|---|---|---|---|---|---|---|---|---|---|---|---|---|---|---|---|---|
| (years) | O | E | O/E | O | E | O/E | O | E | O/E | O | E | O/E | O | E | O/E | |
| < 5 | 0 | 2·99 | 0·00 | 2 | 1·45 | 1·38 | 0 | 1·05 | 0·00 | 0 | 0·95 | 0·00 | 2 | 6·45 | 0·31 | 2·33 |
| 5–9 | 3 | 3·37 | 0·89 | 3 | 1·25 | 2·40 | 1 | 1·04 | 0·96 | 4 | 1·01 | 3·95 | 11 | 6·67 | 1·65 | 6·62 |
| 10+19 | 4 | 7·14 | 0·56 | 4 | 2·21 | 1·81 | 4 | 1·75 | 2·29 | 0 | 1·86 | 0·00 | 12 | 12·90 | 0·93 | 0·00 |
| > 20 | 3 | 4·84 | 0·62 | 0 | 1·87 | 0·00 | 2 | 0·74 | 2·69 | 4 | 2·33 | 1·72 | 9 | 9·78 | 0·92 | 0·43 |
| $\chi^2$ for trend | | | 2·59 | | | 0·07 | | | 3·46 | | | 0·64 | | | 0·01 | |

significant trend ($\chi^2 = 6{\cdot}62$, $P = 0{\cdot}01$) is found for dose in the period 5–9 years from first exposure. However, as this is not reflected in the other intervals, and as carcinogenesis requires a longer induction period than this for most other carcinogens, this trend is almost certainly due to chance. Further analysis, by calendar year at first exposure or duration of treatment, failed to reveal any further trends.

The 142 patients who were examined for skin manifestations of arsenicism suggested an interesting potential dichotomy. As a group their overall mortality (O/E = 29/30·38) and cancer mortality (O/E = 7/6·90) was not remarkable, and was consistent with the remainder of the cohort (Table IV). Within this group, 45% had keratoses, 14% had hyperpigmentation, and 11% were found to have skin cancer. Some patients showed 2 or all 3 signs of arsenicism, and altogether 69 (49%) showed at least one of these signs. The appearance of signs was dose and age related. Patients with signs had higher median doses (672 mg) than those without any signs (448 mg) and this was highly significant when assessed by the Wilcoxon rank-sum test ($P = 0{\cdot}001$). Furthermore 40% of patients with signs had cumulative doses above 1000 mg, which applied to only 22% of patients without arsenical signs. On average, patients with signs were 7 years older and had received their first exposure 6 calendar years earlier. Within the group with exposures > 1000 mg, those with signs

TABLE IV.—*Mortality from all causes and all neoplasms according to the presence of arsenical keratoses, hyperpigmentation and/or skin cancer*

| | | | All causes | | | All neoplasms | | |
|---|---|---|---|---|---|---|---|---|
| | | N | O | E | P (2-sided) | O | E | P (2-sided) |
| Examined for signs of arsenicism (deaths after examination) | + | 69 | 20 | 16·05 | 0·38 | 7 | 3·76 | 0·17 |
| | − | 73 | 9 | 14·33 | 0·19 | 0 | 3·14 | 0·086 |
| | T | 142 | 29 | 30·38 | — | 7 | 6·90 | — |
| Not examined for signs of arsenicism (or deaths before examination) | | 336 (+2706 man-years) | 110 | 130·84 | 0·04 | 27 | 28·85 | — |

were again on average 7 years older and had their first exposure 3 calendar years earlier.

It is of interest that all 7 subsequent deaths from internal malignancy occurred within the group showing physical signs of arsenicism. This excess over an expected 3·76 cancer deaths is not statistically significant ($P = 0{\cdot}17$). The details of these cases are listed in Table V. No deaths from cancer occurred in the group without physical signs (O/E = 0/3·14, $P =$ 0·086).

Within the examined group, two other individuals had cancer mentioned on the death certificate, but not as the underlying cause of death; both were clinically free from cancer when examined. One had cancer of the bladder and was a heavy smoker; he had received 1800 mg of arsenic over 2 months for eczema 15 years before death, and showed no signs of arsenic exposure on examination 9 years before death. The second was a woman whose certified cause of death was a left hemiplegia at age 77; she had a malignant melanoma, and signs of arsenicism (including a basal-cell carcinoma) had been noted 7 years before death. On post-mortem examination, bladder papillomas were also found. She had received 173 mg of arsenic more than 50 years previously for pyelitis.

### DISCUSSION

These data provide at the most only a weak indication that the ingestion of Fowler's solution over long periods leads to any overall increase in mortality from cancer. The 90% confidence limits on the risk ratio (0·70–1·27) and the mean follow-up period (20·3 years) in conjunction with similar findings reported by Reymann *et al.* (1978) indicate that any excess risk for all neoplasms must be small. The most apparent feature was the deficit of deaths from cancer and all causes in patients receiving < 500 mg of arsenic. If this deficit, which was also weak in the series of Reymann *et al.* (1978), can be taken to reflect an incidence of cancer among unexposed individuals with this group of skin diseases, which is below the national average, there may be a significant excess of cancer due to ingestion of arsenic. However, the lack of a dose–response relation above 500 mg, and the inability to find an excess during the period 10 or more years after first known exposure, make this unlikely.

The number of cancers at a few sites was in excess of expectation. A slight excess of lung cancer was evident (Table I) with a suggestion of a dose–response relationship (Table II). However, both these tendencies were weak and far from statistical significance. The one death from malignant skin cancer (of the anus) probably resulted from the use of arsenic. A second death from multiple epitheliomas in a man at age 87 in whom hyperpigmentation and skin cancer had been noted 9 years previously was not included in the formal analysis because we have specifically excluded observations on people over 85-years, but this death was most probably also due to arsenical

TABLE V.—*Cases of cancer after discovery of signs of arsenicism*

| Site of cancer | Sex | Age at 1st known exposure to arsenic | Total dose of arsenic (mg) | Condition for which arsenic was prescribed | Age at examination for signs of arsenic | Interval from examination to death (months) | Keratoses | Hyper-pigmentation | Skin cancer |
|---|---|---|---|---|---|---|---|---|---|
| Chronic lymphatic leukaemia | F | 33 | 336 | Anxiety state | 66 | 30 | X | X | X |
| Bronchus | M | 35 | 25200 | Dermatitis herpetiformis | 51 | 115 | X | X | X |
| Rectum | M | 58 | 4800 | Dermatitis herpetiformis | 66 | 10 | X | | |
| Colon | M | 58 | 346 | Psoriasis | 64 | 97 | X | | |
| Bronchus | M | 54 | 1344 | Psoriasis | 64 | 10 | X | | |
| Bronchus | M | 49 | 3420 | Folliculitis | 70 | 21 | X | X | |
| Pancreas | M | 63 | 1008 | Lichen planus | 70 | 92 | X | | |

carcinogenicity. The slight excess of bladder cancer (3 observed, 1·19 expected) is less likely to be associated with arsenic. These cases occurred in 2 women and a man who had cumulative doses of 504, 963 and 3224 mg beginning 7, 8, and 6 years before death respectively. Unfortunately we do not have detailed histories from which other risk factors could be assessed.

The one cancer death in the category of "cancer of the liver, gall bladder, and bile ducts" was due to a primary in the gall bladder—a type of cancer that has not been associated with arsenic. There were no deaths from primary cancer of the liver.

The most notable positive finding was the prior presence of keratoses among all examined patients who later died from cancer. Whilst the 7:0 split between the groups with and without signs of arsenicism must be partly fortuitous, the suggestion that the appearance of keratoses or hyperpigmentation in cases exposed to arsenic is associated with a higher risk of internal malignancy is in keeping with findings in other less controlled studies, and merits further attention. A biological basis for this phenomenon has been suggested by Bettley & O'Shea (1975), who found that patients with arsenical keratoses and superficial carcinomas had a greater retention of a test dose of arsenic than normal controls.

The lack of cancer in patients without signs is more difficult to explain, and is not supported by other evidence. It is presumably due to chance, but the possibility may be considered that patients who fail to develop signs after exposure to arsenic form a subgroup that is also less susceptible to other carcinogens. It remains possible that some of the contradictory reports on the carcinogenicity of arsenic can be explained by the variability among individuals in their ability to excrete ingested and inhaled doses of arsenic.

We thank Sir Richard Doll for his helpful remarks and Miss Carol Hermon for her expert technical assistance.

REFERENCES

ATKINSON, S. C. (1969) Arsenical keratoses and internal cancer. *Arch. Dermatol.*, **99**, 237.

AXELSON, O., DAHLGREN, E., JANSSON, C. D. & REHNLUND, S. O. (1978) Arsenic exposure and mortality: A case-referent study from a Swedish copper smelter. *Br. J. Indus. Med.*, **35**, 8.

BEAN, S. F., FOXLEY, E. G. & FUSARO, R. M. (1968) Palmar keratoses and internal malignancy. *Arch.* Dermatol., **97**, 528.

BERGOGLIS, R. M. (1964) Cancer mortality in zones of arsenical waters of the Province of Cordoba, Argentine Republic. *Prensa Med. Argent.*, **51**, 994.

BETTLEY, F. R. & O'SHEA, J. A. (1975) The absorption of arsenic and its relation to carcinoma. *Br. J. Dermatol.*, **92**, 563.

COWLISHAW, J. L., POLLARD, E. J., COWEN, A. E. & POWELL, L. W. (1979) Liver disease associated with chronic arsenic ingestion. *Aust. N.Z. J. Med.*, **9**, 310.

DOBSON, R. L. & PINTO, J. S. (1966) Arsenical carcinogenesis. In *Advances in Biology of Skin.* (Eds Montagna & Dobson). New York: Pergamon. **8**, 237.

DOBSON, R. L., YOUNG, M. R. & PINTO, J. S. (1965) Palmar keratoses and cancer. *Arch. Dermatol.*, **92**, 553.

EVANS, S. (1976) *Arsenic and Cancer.* M. D. Thesis, University of Liverpool.

FROST, D. V. (1977) The arsenic problems. *Adv. Exp. Med. Biol.*, **91**, 259.

HILL, A. B. & FANNING, E. L. (1948) Studies in the incidence of cancer in a factory handling inorganic compounds of arsenic. I. Mortality experience in the factory. *Br. J. Indus. Med.*, **5**, 1.

HUTCHINSON, J. (1888) On some examples of arsenic-keratoses of the skin and of arsenic-cancer. *Trans. Pathol. Soc. Lond.*, **39**, 352.

LANDER, J. J., STANLEY, R. J., SUMNER, H. W., BOSWELL, D. C. & AACH, R. D. (1975) Angiosarcoma of the liver associated with Fowler's solution (potassium arsenite). *Gastroenterology*, **68**, 1582.

LEE, A. M. & FRAUMENI, J. F. (1969) Arsenic and respiratory cancer in man: An occupational study. *J. Natl Cancer Inst.*, **42**, 1045.

MABUCHI, K., LILLIENFELD, A. M. & SNELL, L. M. (1980) Cancer and occupational exposure to arsenic: A study of pesticide workers. *Preventive Med.*, **9**, 51.

NEUBAUER, O. (1947) Arsenical cancer: A review. *Br. J. Cancer*, **1**, 192.

OTT, M. G., HOLDER, B. B. & GORDON, H. L. (1974) Respiratory cancer and occupational exposure to arsenicals. *Arch. Environ. Health*, **29**, 250.

PRYSTOWSKY, S. D., ELFENBEIN, G. J. & LAMBERG, S. I. (1978) Nasopharyngeal carcinoma associated with long-term arsenic ingestion. *Arch. Dermatol.*, **114**, 602.

REGELSON, W., KIM, U., OSPINA, J. & HOLLAND, J. F. (1968) Hemangioendothelial sarcoma of liver from chronic arsenic intoxication by Fowler's solution. *Cancer*, **21**, 514.

REYMANN, F., MOLLER, R. & NIELSEN, A. (1978) Relationship between arsenic intake and internal malignant neoplasms. *Arch. Dermatol.*, **114**, 378.

ROBSON, A. O. & JELLIFFE, A. M. (1963) Medicinal arsenic poisoning and lung cancer. *Br. Med. J.*, **ii**, 207.

ROCKSTOH, H. (1959) Zur Äetiologic des Bronchialkrebses in arsenverarbeitenden Nickelhutten. *Arch. Geschwulstforsch.*, **14**, 151.

ROTH, F. (1956) Ueber die chronische Arsenvergiftung der Moselvinzer unterbesonderen Berücksichtgung des Arsenkrebses. *Z. Krebsforsch.*, **61**, 287.

SOMMERS, S. C. & MCMANUS, R. G. (1953) Multiple arsenical cancers of skin and internal organs. *Cancer*, **6**, 347.

TOKUDOME, S. & KURATSUNE, M. (1976) A cohort study on mortality from cancer and other causes among workers at a metal refinery. *Int. J. Cancer*, **17**, 310.

YEH, S. (1973) Skin cancer in chronic arsenicism. *Hum. Pathol.*, **4**, 469.